# Social regulation activities in end-of-life: a qualitative study on completion of advance directives in Swiss nursing homes

**DOI:** 10.1186/s12904-020-00562-9

**Published:** 2020-04-23

**Authors:** M. Droz Mendelzweig

**Affiliations:** Institut et Haute Ecole de la Santé La Source, Avenue Vinet 30, CH - 1004 Lausanne, Switzerland

**Keywords:** Advance directives, Nursing homes, End-of-life, Dementia, Regulation theory

## Abstract

**Background:**

In Switzerland as in many countries, steady trend is observed in nursing homes to promote writing of advanced directives (ADs). Implementation of ADs reflects the rise in public concern for the persons’ right to self-determination and informed decision. The issue of end-of-life conditions is particularly acute in situations with dementia. This article investigates how ADs interventions in nursing homes strive simultaneously to behave in line with the principles of care ethics and with the intention to respond to legally binding instructions. Healthcare to dying residents with dementia in nursing homes is interpreted in light of the Regulation theory.

**Methods:**

Nursing home palliative care reference nurses were contacted through questionnaire. One hundred twenty-one addresses were reached, 69 responses were collected, giving a response rate of 57%. In order to deepen the understanding, 10 semi-directive interviews were conducted in 10 different nursing facilities with 12 palliative nurses.

**Results:**

Presently, Swiss nursing homes are lacking a model of AD suitable to people with dementia. The study sheds light on dissimilarities in the purpose assigned to ADs’ procedure in the different facilities. Discrepancies in end-of-life care practices reveal more the influence of structural and organisational devices specific to each setting than conflicting views on end-of-life care principles. We analyse the interpretation of the Law and its implementation in the participating NHs as compromises that could be accounted for as a form of social regulation.

**Conclusion:**

Dementia accentuates the uncertainty inherent to end-of-life trajectories. The implementation of standardised procedures aimed at collecting the wishes of the person deprived of his or her discernment is source of dissonances with regard to the multiple interests involved in these care situations. In this context, the drafting of ADs during end-of-life care in NH correspond to new normative constraints requiring new collective regulation actions.

## Background

Our study was conducted in 2014–15, 1 year after the implementation of a revised article in the law governing the protection of vulnerable adults in Switzerland and a dozen years after the implementation of a palliative healthcare program decided by the Vaud Canton Public Health services. Assuming that these two resolutions were likely to impact end-of-life care practices, we sought to examine how these changes were reflected in the practices of institutions caring for people with dementia.

Ever since 2013, advance directives (ADs) and therapeutic respondents have been enforced by a binding legal value. The main purpose of this legal framework is to strengthen the right of individuals to self-determination, particularly with regard to medical treatment. The concept of ADs allows adults to draft guidelines and to appoint a trusted person to ensure that their wishes are respected in the event of a subsequent loss of their capacity for discernment. The Swiss law designates this role as therapeutic representative, usually referred to in the English literature as a proxy or surrogate decision-maker.

Although ADs are meant only for the organization of care in particular situations, a tendency to refer to this instrument in the context of end-of-life care can be observed, particularly in end-of-life situations with dementia in nursing homes (NHs). In these contexts, ADs are not treated solely as wishes for medical treatment that patients may have expressed BEFORE entering the NH but as indications for the end of life, to be drawn up during the stay if they did not exist prior to the individual’s entry into the institution. Clearly, advance health decisions in prevision of a loss of decision-making capacity is of concern for most persons entering an NH. Very often, in their case, this situation conflates proximity with end-of-life. However, formally, ADs are not an instrument intended for the management of end-of-life. Therefore, it should be acknowledged that considering ADs as a tool for giving instructions for end-of-life and not for decision-making capacity is a common misunderstanding. However, the intent of this article is to show that this misunderstanding allows ADs to be addressed and used as a part of a broader “care planning” and that this is consistent with the ethics of end-of-life care. This study depicts how NHs use AD forms or forms adapted from existing AD in order to support patients’ and proxies’ thoughts on life-threatening events. The study shows that, in the course of the care process, it is very difficult to draw a clear delimitation between the anticipation of the loss of discernment and prevision of one’s end-of-life. As one leads to the other, we observe that NHs produce ADs when residents have not drafted them before entering the institution. Such a situation is generally the case. Where tools designed for a given scope are used to serve another purpose, we analyze this permutation as the action of a social regulation mechanism. We explain why we consider that kind of pragmatism to be consistent with the spirit of the law (but not the letter).

Our study indicates that presently, a model of ADs suitable for people with dementia is lacking. The analysis developed in this article is that actions taken to implement care planning for NH residents with dementia support nursing ethical principles as the spirit of the Law despite their distance from the letter of the Law. The study observes a gap between the principle of ADs as conceived in the legal framework and in the documents drafted in the course of end-of-life healthcare for persons with dementia. The working procedures observed show that nurses in charge of healthcare for dying persons with dementia are actively involved in translating legal norms into working rules. It can be considered that their commitment to implementing care ethics is driven by the imperative to give voice to the residents’ wishes. Although dissonances have been observed between the normative AD model and the AD records drafted in NHs, they are not necessarily opposed.

Due to the high proportion of residents currently with dementia in NHs, we assumed that the rarity of ADs is a matter of concern for NH management. In this study, we did not focus on assessing the decision-making capacity that preceded the resident’s entry into the NH, choosing to focus solely on the care provided to these persons at the time of their end-of-life at the institution and, more specifically, on the place given to this assessment in the midst of the end-of-life process.

In the study presented in this article, it appears that the use of ADs remains extremely scarce among the elderly before moving to the NH. In line with that finding, it was observed that the implementation of the article of law has motivated the implementation of decision-making tools and that the drafting of these instruments was occupying the staff in charge of palliative care from the time the new resident is admitted until her/his death.

Switzerland being a confederal state, each Canton has cantonal self-rule in administrative affairs. To implement the political intention to transform accompaniment in dying into a professional skill, the Vaud public health office adopted, as early as 2007, a cantonal program for the improvement of palliative care services in nonhospital settings. Nurses in charge of palliative care have been appointed in each NH of the Canton. In our study, we note that the cantonal palliative care program – designed to promote implementation of self-determination principles – lacks uniformity. There are differences among NHs in the training received by those appointed to the palliative function, in the levels they occupy in the institutional hierarchy, in the level of training required for the position, in the recognition given to their function, and in the institutions’ expectations of them.

Indeed, the array of NHs’ responsibilities in decision-making covers highly sensitive issues in terms of clinical, ethical and legal impacts [1]. The ethics of end-of-life care, to which palliative nurses are committed, often encounters inconsistencies in procedural, social or moral logics. One aspect is the tendency to make the establishment of ADs *mandatory*, while according to the Law, ADs are an *option* given to individuals seeking in their own right to formalize their acceptance or refusal of future treatments in case of incapacity *at a later stage*. Another aspect is the difficulty healthcare professionals and residents experience in understanding precisely what ADs do or do not entail [2]. Given that, at present, the proportion of residents with dementia in NHs is estimated at between 60 to 70% [3], it is obviously important for end-of-life care providers to possess the means to respond to their residents’ wishes. We observe that the entry into force of the revised article of law has also given impetus to the formalization of procedures in favor of the drafting of ADs. If the first intention in the revision of the law was to limit medical authority in the healthcare process, one can notice the emergence of a regulation process effect, leading NH personnel to adapt their working procedures.

However, the establishment of ADs promoted at present by NH management refers more to the rise in public concern for self-determination than to explicit requests coming from the residents or from their relatives to formalize their terminal wishes (on this topic, see also [4]). End-of-life conditions occupy an important place in this tendency and, particularly in our aging societies, concern for end-of-life with dementia. We assume that, given their role at the intersection of the healthcare and the social fields, healthcare professionals working in residential care settings find themselves in a position to fulfill a pivotal function, qualified as a “nursing brokerage model” by Jeong, Higgins and McMillan [5].

The aim of this study was to discover to what extent end-of-life care provided to NH patients is consistent with the principles of the Law and to understand the reasons for any discrepancies with the Law. More specifically, if the activities linked to ADs observed in NHs are mostly concerned with formalizing procedures that have already been applied, we were interested in understanding why, then, NHs find it necessary to organize them differently. Hence, we strive to investigate the status of the AD files established in NHs and to determine the purpose assigned to them by the different institutions. Do such documents have a purely utilitarian role? Are they substitutes to address the lack of formal guidelines used prior to the occurrence of the dementia disorders, or do these documents legally correspond to ADs? Without deciding on the legal question, which is not our field of study, we focus on the practical consequences of this normative framework for nursing ethics. We suggest that the regulation theory provides a useful interpretative framework for developing an understanding of end-of-life care confronted by residents with dementia facing death.

The regulation theory, drafted by Jean-Daniel Reynaud in the 1950s, provides our theoretical framework for developing an understanding of how social actors coordinate their collective actions and grant them authority through the establishment of rules. In light of the theory of regulation, we have analyzed the drafting of ADs in the context of NHs as new normative constraints requiring new collective regulation actions. In this article, we will describe how NHs integrate this new provision into their care and how they interpret it with respect to their residents with dementia at the end of their lives.

### Studies investigating the use of decision-making tools in NHs in support of patients with dementia

Even before the start of our field survey, we were aware of the scarcity of the use of ADs among elderly people entering institutions. During the time of our study, this topic of advance care was addressed by the Swiss Academy of Medical Sciences, which initiated a research program on the issue. The study on which the present article is based was funded within this specific research program. In 2016, the Swiss Federal Office of Public Health adopted measures aiming to strengthen the anticipation of care in situations where need is felt most urgently, in particular, in NHs accommodating people in the last months of their lives. In this context, advance care planning (ACP) is designed as a “prospective instrument for planning, preparation and decision-making and to avoid unnecessary hospitalization” [6]. In this regard, it is stipulated that “the quality and importance of ADs and incapacity mandates must be reinforced to allow for a coordination of treatment and follow-up of patients better adapted to their needs”. In line with this position, the Swiss National Ethics Commission considers that ADs should be treated as a global ethical framework, thereby admitting that the application of this framework must ensure particular adjustments to each singular case [7]. Our literature review confirmed that the promotion of ADs as part of an advance care planning approach is not unique to Switzerland.

A study on the legal framework for ADs in the EU member states stresses the ethical duty to always assess capacity in each individual case [8]. In a systematic literature review, Flo et al. [2] recommend viewing ACP as a process rather than as an administrative formality resolved once and for all. From the same perspective, the Leadership Alliance for the Care of Dying People [9] points out the importance of open communication with patients and families concerning the inherent uncertainties involved in end-of-life situations. It should therefore be concluded that ACP has a broader meaning than ADs, which are to be seen as a formal support available to patients and caregivers to enable the anticipation of care.

The Liverpool Care Pathway (LCP) model in particular has generated extensive studies on the implementation and limits of ACP [10]. Sharp et al. [11] have conducted a review of literature on the attitudes of the public and healthcare professionals towards ACP discussions with frail and older people. They found several authors noting the difficulty elders have in making well-informed decisions before illness occurs. Moreover, investigating healthcare professionals’ attitudes towards end-of-life care conversations, they also found that, although the medical staff recognize such discussion as part of their professional responsibility, very often, workload pressures and uncertainty over prognosis inhibited the initiation of these discussions.

Whereas the literature underlines strong correlations between completion of ADs and reduced use of invasive medical procedures and hospitalizations at the end of life [12], few studies have examined the issue from the global point of view of the adoption of the legal AD principle by the elderly public. Thus, a survey performed in ten different American states in 1993, just 3 years after the implementation of the Patient Self-Determination Act (PSDA), shows very modest changes in the documentation of living wills in NHs. Increases have been noticed in “Do Not Resuscitate Orders” (DNRs) and in “Do Not Hospitalize Orders” (DNHs) and a stagnation in orders to forgo artificial hydration and nutrition. According to the authors, these features are indicators of trends already underway prior to the PSDA implementation [13]. The interesting point is the debate initiated since then about the appropriateness of ACP for NH residents. Some years later, the situation does not seem to have changed drastically, either in terms of the adoption of the principle or of the homogenization of procedures. A study conducted in Australia found very low levels of formal ADs among NH residents and no clearly identified process for medical decision-making [14]. Another example from Germany indicates that, despite the introduction in 2009 of an Advance Directives Act, there has been a very low uptake of decision-making documents in NHs [15]. No more than 11% of the residents have a personal AD, but these ADs were also found to be too ambiguous to indicate what treatments would be accepted or not in case the patient loses his/her capacity of consenting to treatments. Barclay et al. [16] come to quite similar conclusions by recognizing uncertainty as the main feature that describes residential care home residents’ trajectories to death.

In light of this inevitable confrontation with complex nursing ethics issues, we undertook this study on how the revision of the Law affects end-of-life care in Swiss NHs.

## Methods

This study is based on a comprehensive approach using a theoretical framework that draws upon ethics of care theories [17] and on the regulation theory. To obtain a broad view of how the revision of the Law influences the determination of end-of-life care for residents with dementia in NHs, we opted for the Panoramic Situational Contextualization Analysis method [18]. This method allows us to both obtain a Canton-wide overview of the effects of the revision of the Civil Code on care practices for residents with dementia and further investigate concrete aspects of the implementation of the care principles through qualitative interviews.

This method’s design includes three stages. The initial phase was the collection of data. We performed data collection with the help of a questionnaire addressed to the healthcare professionals designated as palliative care resources in their respective place of work. Since the introduction in 2012 of a palliative care program specific to the Canton Vaud, all the care facilities for older people in the canton employ at least one palliative care reference person.

Drawing on the definition of ADs in the article of law, the questionnaire aimed to determine how NHs’ intention to apply the legal guidelines for ADs is reflected in the care practices for people deprived of their discernment. The study covered the following topics: training pathways in palliative care of the respondents, their institutional guidelines for end-of-life care, the nature and content of the wishes expressed by residents regarding their end-of-life care, and the proportion of those who possessed ADs on arrival. Open questions covered institutional arrangements for the collection of residents’ wishes (documents used, subjects addressed, compilation methods, data processing modes) and short descriptions of situations concerning end-of-life decision-making. Ultimately, respondents’ views on the adequacy of their care practices with regard to the wishes of the residents were required.

Based on a first analysis of data, the second phase was the elaboration of a case study. Ten palliative care reference individuals were chosen based on their mention in their responses to the questionnaire of the existence of specific tools used in the institutions. Ten NHs with contrasting types of care for dying residents with dementia were chosen.

The third phase of the method consisted first of conducting qualitative interviews with each of the ten palliative care referents selected for the sample. The data collected from these interviews were used to create a picture of the different institutions’ visions of how to implement the article of law. This framework then allowed us to conduct an analysis based on a comparison of the situations described by the actors. (Table 1 here).

**Table 1:** Data organization table for qualitative analysis, following the Panoramic Situational Contextualization Analysis Method

Topics	Key meaningful communications	Elements “induced” by the description			
Actors					
		Stakes, challenges End-of-life with dementia	Norms Internal organization of the NHs, in particular the compartmentalization of units; strict adherence to deadlines and procedures; the dominance of channels and disciplinary authorities	Positions Intelligence of the actors in care situations	Relations Awareness of the actors’ mutual dependency on one another in the performance of their duties
Actor A					
Actor B					
Actor J					

At the launch of the study, all the NHs operating in the canton (*N* = 121) received an email from the research team asking for their respective management’s agreement to participate in our study. Responses to our request provided us with the individual email addresses of 77 appropriate persons to answer our questionnaire (see additional file: inquiry questionnaire) as well as 44 institutional addresses.

Each respondent was invited to reply to the questionnaire through a link connecting to a computer survey program (SphinxOnline). The questionnaire was developed in collaboration with an external subcontractor professionally skilled in public health quantitative surveys, to whom the research team assigned the task of addressing the questionnaire and collecting the answers. The comprehension of the final instrument was pre-tested with 10 volunteers before addressing it to the participants via this office. The replies received were also collected via this subcontractor, who returned the answers to the research team in anonymous form. The questionnaire included a point about participants’ consent to communicate their personal data to allow the researchers to contact them for further participation in semistructured interviews. The questionnaire remained available online for 3 months. The total response time to the questionnaire was estimated at 1 h. Participants were able to interrupt and resume their response work at any time during this period. To ensure the reliability of the responses to the questionnaire, it was suggested that participants refer to real cases of residents with dementia accompanied at the NH during the last 12 months preceding the survey. The consultation of the residents’ files used as examples for the answers was recommended in cases where questions required clarification of details. The items dealing with specific aspects related to the organization of end-of-life care were treated as single-choice questions, or closed-ended, or multiple-choice questions. Questions seeking respondents’ opinions on whether the residents’ wishes were being respected were formulated as a Likert scale from very satisfied to very dissatisfied or from strongly agree to disagree. The responses to the questionnaire contained almost exclusively nominal qualitative variables that were processed using multivariate descriptive analysis methods.

Sixty-nine participants returned the questionnaire at the end of the period, for a response rate of 57%, as long as we consider that each NH on the list provided a single response (which is not necessarily the case; some responses were probably the result of team consultation, although respecting our respondents’ anonymity prevents us from clarifying this aspect further). A total of 73% of the respondents were women (*N* = 51), all of them involved in palliative care for 2 years and more. The responses to the questionnaire, expressed in percentage terms, had a lower statistical value due to a considerable rate of nonresponse to several questions. The datasets generated during the current study are available in the FORSbase repository, https://forsbase.unil.ch/project/study-public-overview/15410/0/.

The ten in-depth semistructured interviews, lasting 1 h each, were conducted with 12 nurses in their workplace: one male and eleven females with professional experience in geriatric settings ranging from 20 to 35 years. The three members of the research team (two university researchers trained in qualitative research, one psychologist and one anthropologist, and a clinical psychologist, a member of the palliative care unit of a regional hospital) shared the responsibility for conducting interviews. Each of the three researchers took part in filling in the panoramic chart for the study of situations after conducting a group debriefing on their respective interviews.

**Fig. 1:**
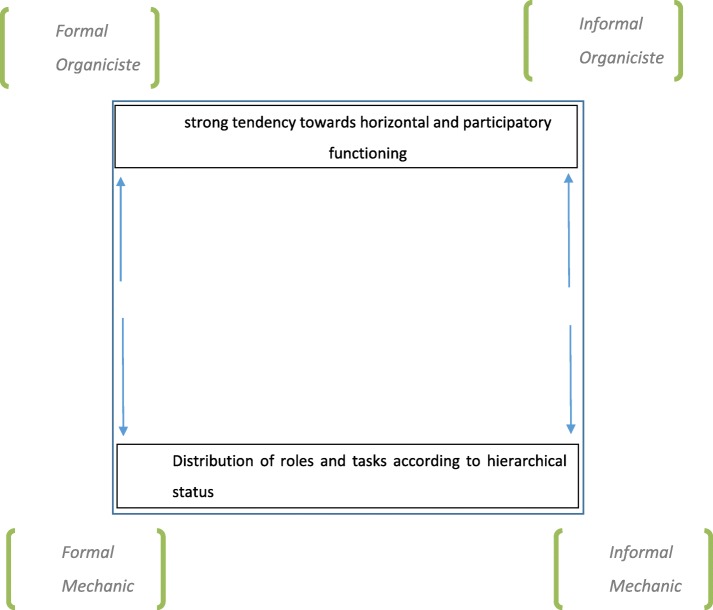
Typology of ADs management according to the organizational pattern of NHs

The situations were compared by ordering the data in the chart (Fig. 1). On this basis, four typologies of institutional management were identified: “formal” and “mechanistic” NHs; “informal” and “mechanistic”; “formal” and “organicist”; and “informal” and “organicist” [19] (Fig. 1 here). The “mechanistic” type is characterized by rather formal and explicit practices, either supported by a strong hierarchical framework (“formal”) or a more spontaneous management structure (“informal”); conversely, “organicist” situations were characterized by horizontal approaches for the care of dementia residents in end-of-life, recruiting support among all the staff present, either following a rigorous planning approach (“formal”) or one based on each specific situation.

The findings described hereafter are founded on a synthesis of data stemming from the questionnaire and from the qualitative interviews.

## Results

The results of the questionnaire confirm the lack of ADs among elderly persons entering NHs. From the hundreds of residents with dementia admitted to NHs during the year preceding the study, only 60 belonging to four different institutions were mentioned as having established their ADs before entry. In other words, most of the AD documents NHs utilize refer to documents drafted during the residents’ stay in the NH. These documents were not written at the initiative of the person at stake but are instead the result of a management decision, making these documents part of the nurses’ duties.

Replies to the questionnaire as well as the qualitative interviews both show that all NHs foster specific organizational arrangements for end-of-life care. In each of them, collection and formal compilation of data about the residents’ and their families’ wishes are central activities involving a large number, if not all, of the staff members from the very moment of the entry of a new resident to the institution. The interviews make it possible to deepen the understanding of the links between aspects related to the organization of end-of-life care and the global institutional management. An interesting parallel can be drawn between the progress of specific arrangements for ADs in institutions and the position occupied by the professional in charge of palliative care. Thus, it appears from the interviews that the institutional framework is proving to be a determining factor for the deployment of end-of-life care practices. Differences are observed in the way the respective NH managements encourage their teams to ensure that ADs are drawn up. Two types of management emerge from the analysis of the interviews thanks to the support of our chart (Table 1): those that enforce a rigid and formalized procedure and those that enforce a more informal, collective procedure. The evidence indicates that the reasons for enforcing a formalized versus an informal procedure are due to management practices in the institutions rather than to diverging ethical principles related to end-of-life care or to clinical imperatives. The former is characterized by its hierarchical organization. The duties related to the drafting of ADs are given to staff members in charge of palliative care and are placed under the chief nurse’s responsibility, while the latter implements a horizontal management of end-of-life procedure and adopts a marked tendency to involve all employees known for their proximity to the resident, drivers and housekeepers included.

The structural stability of the NH at the time of the survey appeared to be a decisive factor in the distinction between these two categories of setting. The differences observed between institutions were based more on issues related to their management than on differences in their visions of care. Thus, it is aspects such as expertise of the teams, the recognition of their professional skills, the trust relationship within the teams and between them and the management of the home that drive NHs to fall into one or the other of these two categories. The institutions that demanded the most rigor in terms of timeliness in drafting the ADs and their contents were also those where there were vacancies at the executive level. In contrast, the second category had more stability in their internal organization; the persons in charge of end-of-life healthcare treatments had usually been in the workplace for many years and enjoyed assertiveness in their relations with the hierarchy and with staff members.

In either category, during the interviews, nurses’ replies to the questions related to the implementation of the end-of-life treatments drew a distinction between “technical care and end-of- life decisions” on the one hand and “human relationships close to death” on the other. This classical dichotomy between the relational and technical characteristics of healthcare activities exemplifies conflicting views, even though the two aspects are intimately interwoven in the description of the related activities.

### Data collection on end-of-life wishes

Results of the questionnaire confirm that all NHs require a document containing the residents’ wishes for the end of their lives at the end of the first month of entry. Forty-three of 50 replies to the questionnaire acknowledged the use of specific forms for this drafting and having at their disposal specific guidelines to follow while performing this task. Thanks to the semistructured interviews of phase 3, we were able to observe that the implementation of the AD forms and the procedures to fill them were still in progress in most cases. Differences in their respective objectives were also observed: for some healthcare teams, the purpose of the procedure was strictly informative, while for others, it was intended to be a support for terminal treatments. Consequently, NHs differ in the content of their AD files. No unanimity could be decided on what the minimal information required for the AD records should be. For some, the level of detail required is insufficient; for others, entering too deeply into details is an open door to the risk of being too directive and of limiting in advance the decision-making process. Questions were raised during the interviews on the relevance of including in the AD form items related to quality, values, and life principles as well as to funerals. Respondents’ positions differed radically according to the case to which they referred in their answers. The general opinion on this topic stemming from the semidirective interviews was that, beyond any implemented guidelines, behaving appropriately in end-of-life situations always depends on the quality of relationships established with the dying person and with her/his relatives.

Perceptible tension was noted between introducing systematization to data collection for end-of-life treatments out of respect for the dying person’s self-determination and the relational feasibility of this data collection. All qualitative interviews addressed the sensitivity required to discuss thoughts regarding death with the resident and with her/his relatives. Respondents claimed that the right time to do so rests on the familiarity developed in each situation. The general opinion in favor of a gradual approach contradicted requests from NHs’ management to have the AD files filled in by the end of the first month at the institution. These aspects were developed only in the interviews.

One section of the questionnaire raised the topic of staff members’ openness to reconsideration of the advance care plans according to the residents’ state of health. Forty-three of 45 replies were affirmative, basing the changes introduced in care planning principally on requests stemming from the relatives. To a lesser extent, such changes were also based on indications coming from the resident herself/himself or from the physician. In accordance with these answers, semidirective interviews emphasized the uncertainty associated with any end-of-life process. Palliative nurses called for adopting a critical stance towards overly detailed written directives and defended the necessity of freedom to adapt advance care plans to the singularity of each situation. Preference was given to maintaining close contact with the dying person and with her/his relatives until the death of the resident. One experienced chief nurse advocated the right to oppose the Law, arguing that any kind of rigidity in the respect of the administrative and legal procedures would contradict the essence of end-of-life care principles. Dementia at the end of life was, in this respect, particularly relevant to the development of a critical distance towards ADs. For these experienced nurses, communication with persons deprived of their discernment is a professional skill, hence their demand to maintain an ongoing dialogue with the dying person until her/his death as well as with her/his relatives. Such communication is why these nurses doubt the relevance of a standardized document.

## Discussion

### The role assigned to ADs in NHs

This study has highlighted different duties assigned to ADs in NHs.

With the aim of complying with the revised article of law on the protection of adults, NHs encourage nurses in charge of palliative care to emphasize the formalization of data collection related to the residents’ and their relatives’ wishes for the end of life. Through these managerial procedures, NHs seek to meet societal expectations regarding the respect of patients’ self-determination. The drafting of documents designated as ADs during the resident’s stay in the institution is intended to address this normative impulse. However, how to implement this process differs among care providers. We have observed two dominant trends.

In one approach, the records are treated as legal documents. These documents bear the name by which the Law designates guidelines aiming to provide information on the medical treatments the person desires or refuses in the case of loss of the capacity to make her/his decision. These documents are dated and signed by the protagonist or by her or his therapeutic representatives as if they were legally binding. In the other approach, these documents are considered as if they were no more than care provisions to refer to for decision-making support during the end-of-life care process.

What may explain the different ways of complying with the Law in NHs is that AD records are documents that exceed the single legal remit. Such records fulfill a threefold function: the respect of the principle of patient self-determination, assistance in the event of a dispute with the residents’ relatives, and support of the care plan for the healthcare teams. This multipurpose nature explains the generalization of AD drafting procedures within NHs: both the institutions’ management and the healthcare teams find them of interest.^1^ However, no institution seems to have found the ideal formula between the option of a short form and one that asks for details on the individual’s end-of-life wishes. Nurses face certain difficulties in reconciling normative requirements with the reality of the individuals for whom they care. The information they are asked to collect requires them to coordinate with the dying person and with her/his relatives; doctors are also sometimes involved. The temporal dimension is not simple, since more often than not, gathering information as soon as possible enters into conflict with taking the time to preserve sensitivities. The intersection of the Law, institutional rules and planned care procedures requires regulatory activity when incorporating the normative framework.

Indeed, AD forms filled in while caring for end-of-life residents do diverge, to some extent, from the article of law on the protection of adult persons. These records often combine administrative data with medical data, evidence collected while caring for the dying person with information received from relatives. Moreover, they juxtapose clinical data with personal narratives and healthcare provisions with considerations concerning values, sensitivities, comfort, and tastes. Nevertheless, in our view, these procedures follow the same ethical principles as those underlying the article of law. We agree with the Swiss National Ethics Commission [13], which considers that ADs should be treated as a global ethical framework. The application of the latter requires adjustments to each singular case. Thus, more legal rigor in the creation of such documents would overlook the ethical intention of this specific tool. Instead, we suggest that ongoing communication with the dying person and with her/his relatives until death, as it is practiced in the NHs involved in this study, can be considered as a caring and ethical way to face the uncertainty that often marks the trajectory to death [14]. Even if, as observed, AD forms do not strictly correspond with the legal format, they nonetheless reflect an awareness of all views, those collected while caring for the residents, those expressed by relatives and those based on palliative healthcare norms. Such documents promote ongoing exchanges between members of care teams and between them and the dying person and her/his relatives. The drafting of these documents can be viewed as a way to render effective the norm that asks healthcare professionals to be aware of the patients’ wishes and to respect them.

It appeared to us that these working procedures had a secondary benefit: that of promoting cooperation between the members of the care teams and the resident’s entourage. Ultimately, the consensus-building process is of more importance than the document itself. It seems to us that this approach of continuous adaptation of the objectives of care to singular conditions is akin to that known as Advance Care Planning [20]. This care model has arisen as a reaction to the issue of uncertainty in end-of-life situations, particularly in those marked by dementia. Through its pragmatic, relational approach, this model aims to overcome the shortcomings of an overly rationalistic view of self-determination for end-of-life care.

### Regulation theory and its utility in the debate about ADs

Drawing on a sociological study related to how institutions, organizations, economic life, working relations and training evolve and interact, Jean-Daniel Reynaud developed as early as the 1950s a global theory about regulation mechanisms. This theoretical framework has evolved into an avenue of research inspiring researchers from different disciplines in the social sciences and humanities. The main premise of his “theory of social regulation” [21] is that rules apply constraints to social actors. Hence, social reality consists in the exercise of this constraint. Adhering to a firmly pragmatic approach, Reynaud considers the confrontation with social constraints as a matter of regulatory activity. However, constraints are not seen as obstacles; on the contrary, for Reynaud, constraints drive collective action: “cooperation could not occur without normative constraint” [22]. In other words, regulatory activities are the very essence of social reality. To function, laws and rules must have operative virtues (“But in all cases, the legitimacy of those rules is procedural, not substantial: rules are not made legitimate by the values that may or may not underlie them; what makes them legitimate is the procedure that allowed for constructing them” [22]). To do so, regulatory activities must be combined with procedures, arrangements, agreements, and common rules that make them operational. In this sense, regulatory activities produce practical effects, which are sought by the social actors.

Social actors create rules and ensure their continuity: Reynaud calls this “autonomous regulation”. These actors do so not only out of fear of sanctions – designated by Reynaud as “regulation of control” [23] – but also because rules should provide them with satisfactory balance. In this respect, initiatives taken by social actors aiming to manage possible incoherence in social life give rise to social rules [24]. Collective regulation activity is a process that gives way to new rules. However, the transformation of rules implies constraints to legitimacy and of effectiveness in a nonstable universe [24]. This process is defined by Reynaud and Richebé [22] as “ordinary normativity”. For this scholar, issues linked to legitimacy explain why the articulation of the creation of new rules and management of the constraints are marked by phenomena of inertia, weariness, and individualistic logics and gives growing space to anomie [25].

The regulation theory is relevant for all sectors of human activities, including the legal sector. For Reynaud [26], legal rules are a kind of social rule, since to function they must combine with other types of rules. Being always in situations of interdependency, social actors must interact, confront one another and conduct exchange. Social actors must thus find common rules to handle disputes, leading them to social exchange. Rather than considering the law as a normative and stable entity, meant to endure, regulation theory invites us to address it as a more or less structured body of rules, norms and standards. In this regard, Jeammaud [27] points to the tendency to confuse legal norms with rules of law. For this author, it is important to consider to what extent norms and rules assume the role of ideal models and to what extent they refer to patterns of behavior and to observable regularities. We may wonder whether these rules, norms and standards meet requirements or whether they refer to evidence taken as references.

This array of thought tends to consider the law as not limited to a legal order placed under the authority of the State but rather as a set of rules that derive their normativity and their juridical status from collective action developed within institutions such as commercial companies, professional groups, health structures, etc. Based in his interest in procedural rationality, analysis of coordination and collective devices, Reynaud understands collective action as the swarming of micro-initiatives, initiatives stemming more from the social actors’ commitment to the construction of a collective utility than from individual utility calculations or terms of contract [22].

Without going as far as stating that Law lies wherever there is normativity, Jeammaud stresses the interest of viewing its relationship to justice as “an attempt to perform justice” (Gurvitch, 1932 quoted by [27]). Hence, from the perspective of the sociology of law, the effective remit of a rule depends on its implementation [26]. The management of basic settings and shaping of new rules are, as defended by de Terssac [24], the very essence of social reality. Following Reynaud and Richebé’s thesis, principles do not precede action but are forged through interaction and negotiation [22].

In our opinion, this interpretative framework is useful for understanding the status of AD records drafted in NHs and for analyzing the role played by healthcare professionals handling these documents in the context of the implementation of the article of law on the protection of adults. Presently, the central position attributed by contemporary societies to the principle of self-determination explains why the AD model has a normative status [28]. In our view, the differences in AD formats observed in institutions for the elderly is not a sign of a loosening in the principle of self-determination but, on the contrary, of concrete ways of transposing it into care practices. The adaptations observed in this study are examples of regulatory activities. Hence, due to the uncertainties inherent to end-of-life, the transposition of the procedures into the practices of care cannot be straightforward. It is a commitment to the ethics of care that leads caregivers to look for ways to adjust the rules. We consider this work as consisting of regulatory activities [29]. Between the requirements to systematize the collection of information on residents’ wishes and the relational conditions of its feasibility, professional caregivers cannot do better than to look for ways to deal with the constraints. By doing so, they produce a collective action that reflects a regulation process. Since end-of-life care can never escape sets of constraints, the ethics of nursing can hardly follow a strictly ordered sequence of operations, and nursing ethics cannot adopt definitive patterns.

### Methodological issues

The study presented here was performed shortly after the implementation of the revised law article. Hence, the lack of homogeneity in the format of the AD documents may be due to the evolving situation. The implementation process may limit the conclusions that may be drawn based upon this study. Furthermore, the perception of the status and format of the guidelines could be different if residents’ or residents’ family members were also interviewed. This approach was not taken in the study presented here.

## Conclusions

An ethical trend is observed in Western societies in favor of autonomy and self-determination. In end-of-life, even more so when end-of-life is marked by dementia, the governing idea rejects excessive medical interventions aiming at the prolonging of life. In this conceptual framework, ADs have gained a normative status in palliative care and in healthcare settings in general. The 2013 revised article of law adopted by the Swiss Civil Code gives a clear expression to this view. However, empirical studies with elderly persons show that the normative standard given to ADs by the Law is not assimilated by the public at large. Elderly persons entering NHs have very seldom established their ADs before their entry to the institution. Finding themselves under pressure to prove compliance with the Law, NHs undertake sustained efforts to ascertain the dying person’s wishes. NHs implement new procedures aiming to collect and record indications about the residents and their families’ wishes. Specific staff members are appointed to this task. The findings were collected through a questionnaire addressing a broad spectrum of issues related to end-of-life care of residents with dementia. The qualitative interviews conducted in this study shed light on the reality of terminal healthcare practices in NHs in Western Switzerland.

Since the reality of social action always implies gaps between the law, rules and standards of conduct, social actors are called to make decisions and often find themselves in a position to make compromises. Legal norms are only a part of a larger set of social rules that palliative care nurses must deal with to satisfy the multiple interests and to respond to the difficulties that arise in end-of-life situations. In this regard, they perform social regulation activities aiming at the implementation of ethical end-of-life norms and rules. Thus, the regulation theory provides useful insights for the interpretation of our findings. This interpretative framework proved to be useful for developing an understanding of nursing ethics confronted by residents with dementia facing death. In light of this theory, the attempts to adapt end-of-life healthcare practices to the principles of self-determination can be understood as regulatory activities.

## Supplementary information

**Additional file 1: Inquiry questionnaire.** This questionnaire is a literal translation of the instrument specifically designed for this research. It was originally drafted in French and used for conducting the inquiry with palliative nurses in the French-speaking Swiss nursing homes context. The purpose of the questionnaire was to collect factual and contextual information on the drafting of ADs in this specific context.

## Data Availability

The authors of the research ensure full transparency of the review process. The search strategy is presented in the method section of this article. All datasets on which the conclusions of the paper rely are available to readers in French on the public repository https://forsbase.unil.ch/project/my-study-list/.

## Abbreviations

ACP: Advance Care Planning
ACDs: Advance Care Directives
ADs: Advance Directives
DNH: Do Not Hospitalize Order
DNR: Do Not Resuscitate Order
EU: European Union
GSFCH: Gold Standard Framework for Care Homes
LCP: Liverpool Care Pathway
MeSH: Medical Subject Headings
NHs: Nursing homes
PPC: Preferred Priorities for Care
PSDA: Patient Self-Determination Act

## Footnotes

1 As we did not seek to obtain the residents’ or residents’ family members’ views on the collection of end-of-life wishes, we do not express their opinions on this subject.
