# Supplementary material for: Social regulation activities in end-of-life: a qualitative study on completion of advance directives in Swiss nursing homes
Source: BMC Palliat Care. 2020 Apr 23;19:57. doi: 10.1186/s12904-020-00562-9 (PMC7181527; doi:10.1186/s12904-020-00562-9)
Supplement: Supplementary file 1 — Additional file 1: Inquiry questionnaire. This questionnaire is a literal translation of the instrument specifically designed for this research. It was originally drafted in French and used for conducting the inquiry with palliative nurses in the French-speaking Swiss nursing homes context. The purpose of the questionnaire was to collect factual and contextual information on the drafting of ADs in this specific context. [file 12904_2020_562_MOESM1_ESM.docx]

**The original of this questionnaire is in French. This is a literal translation of an instrument specifically designed for this research conducted in the Swiss context, which purpose was to collect factual and contextual information. The sampling design for this population was single stage.**

1. **Information on the features of the NH you are working in** (tick the corresponding box)
   1. How many persons are working in your NH (all settings included): 10-20 persons; 20-40 persons; >40 persons
   2. How many beds in your NH: <20 beds; 20-49 beds; 50-99 beds; 100-119; >100 beds
   3. How many residents inter your NH since 2013: <10; 10-19; 20-29; 30-39; 40-49; 59-59; 60<
   4. In which category of care is your NH classified by the Department of Health and Social Action? : psychogeriatric; geriatric; combined mission; long term in patients
   5. In which level of care class is your NH located according to the PLAISIR categories? (OPAS class. The answer must be restrained between 3 and 12):
   6. Among the following training courses, which have been attended by at least one person employed in your NH: CAS^[[1]](#footnote-1)^ in palliative care; CAS in psychogeriatric; DAS^[[2]](#footnote-2)^ in health of aging populations; training course provided by Swiss Alzheimer Association; training course provided by a specific palliative care setting; training course provided in the framework of the implementation of the new Law on the protection of the adult person; other (tick all relevant boxes)
   7. If you ticked the "other" box in the previous point, please specify the other training followed (open answer)
   8. How many persons working in your NH have been trained in one or some of the following training courses: training course provided by Swiss Alzheimer Association; CAS in palliative care; training course provided by Riveneuve palliative care hospice; CAS in psychogeriatric; DAS in health of aging populations; training course provided in the framework of the implementation of the new Law on the protection of the adult person
   9. Does your NH have guidelines for terminal care: yes/no
   10. Can you quote the title or author, or give a reference for these guidelines (open answer)
2. **Information on respondent's professional profile and professional duties** (tick the corresponding box)
   1. You are: woman/man
   2. Your year of birth
   3. What is the highest professional degree you have obtained: community health and care assistant; Red Cross certificate; primary care nurse level 1 or 2; college of nursing 4 years training; bachelor in nursing, postgraduate diploma in nursing, master of science in nursing sciences; postgraduate training followed abroad; other
   4. If you ticked the "other" box in the previous point, please specify (open answer)
   5. What is your current responsibility/position in your NH (open answer)
   6. Have you completed any of the following specific palliative care training courses: palliative care introductory course; CAS in palliative care; CAS in psychogeriatric; Swiss Alzheimer Association training course; Riveneuve palliative care hospice training course; none of these; other
   7. If you ticked the "other" box in the previous point, please specify (open answer)
   8. Have you attended any training on the new Adult Protection Act that came into force on 01-01-2013? Yes/no
   9. If you ticked the "yes" box in the previous point, please specify (open answer)
3. **Provisions made by residents for their end-of-life before their institutionalization** (tick the corresponding box)
   1. How many residents have been admitted into your NH since 2013? <10; 10-19; 20-29: 30-39; 40-49; 50-59: >60
   2. How many of the residents who arrived in your NH in the last 12 months were already in possession of their ADs?
   3. Which of the following aspects have you already seen specified in the residents' advance directives? (only if you have provided an answer >0 to the previous question): health care; religious/spiritual support; administrative aspects; aspects related to family conflicts (tick all relevant boxes)
   4. If you have seen other aspects specified in the residents' advance directives than those listed above, please mention them (only if you have provided an answer >0 to the question 2 above) (open answer)
   5. In general, among the aspects specified in the ADs of these residents, which aspect prevails (only if you have provided an answer >0 to the question 2 in this section): religious/spiritual support; administrative aspects; aspects related to family conflicts; there is not a more prominent aspect, other
   6. Which of the physical care aspects you have seen in the residents' advance directives is most often mentioned? (only those who reported seeing care specified in the residents’ ADs): antibiotics in case of pneumonia; tube feeding in case of refusal to eat; basic care (food, hygiene, comfort); sedation and lucidity; none aspect is mentioned more often than the others
   7. Of the residents who have been admitted since 2013, how many had already appointed a therapeutic representative?
   8. Of the residents who have been admitted since 2013, how many had signed a declaration of incapacity?
4. **Residents’ wishes for their end-of-life and therapeutic representative.** This part concerns the procedures applied in your NH to collect data on the wishes of residents regardless of their cognitive status. Please answer only in relation to cases you have treated since 2013. (tick the corresponding box)
   1. To inquire about the residents' wishes for their end of life, do you use one or more specific documents? Yes/No
   2. What is/are the document(s) used in your NH to inquire about the residents’ wishes for their end-of-life (only people using a specific document): we have designed our own form; Pro Senectute form; Protestant Social Centre form (PSC); Swiss Academy of Medical Sciences form; Cancer league form; Association of Swiss residential and social institutions; We discuss with the resident and provide him with a form to date and sign; other
   3. If you have seen other forms, please specify (only if you ticked the "other" box in the previous point) (open answer)
   4. You have designed your own form to inquire about the residents’ wishes for their end-of-life. The researchers are interested in collecting such documents. Would you agree that our service provider ESOPE will provide your email address to the researchers (completely separate from your answer) so that they can contact you to obtain a copy? (only those who have answer having designed your own form): yes/no
   5. How many ADs have been drafted since 2013 in your NH as a result of the care provided to residents?
   6. Among the following aspects, which were specified in the ADs of these residents? (only those who did not answer 0 to the question 5 in this section): health care; religious/spiritual support; administrative aspects; aspects related to family conflicts (tick all relevant boxes)
   7. Are there any other aspects specified in these residents' advance directives? If so, which ones? (only those who did not answer 0 to the question 5 in this section) (open answer)
   8. In general, among the aspects specified in the advance directives of these residents, which is the most frequently addressed aspect? (only those who did not answer 0 to the question 5 in this section): antibiotics in case of pneumonia; tube feeding in case of refusal to eat; basic care (food, hygiene, comfort); sedation and lucidity; none aspect is mentioned more often than the others
   9. Does your NH formally request the appointment of a therapeutic representative? yes/no
   10. Is the appointment of a therapeutic representative done systematically or on a case-by-case basis as necessary? (only those who have answer yes to previous question): systematic for all residents; on a case-by-case basis as appropriate
   11. Do you use in your NH an "Advance care planning " model in order to remain as close as possible to the resident's wishes for the end of his life? yes/no
5. **Presumed wishes of residents with dementia for their end of life.** This part concerns the procedures applied in your NH since 2013 to collect data on the wishes of residents with dementia for their end-of-life. “Dementia” refers to a person with a clinical syndrome that combines cognitive and behavioral disorders that impair his/her ability to use judgment. Please answer only in relation to cases you have treated since 2013. (tick the corresponding box)
   1. Is it common practice in your NH to inquire informally about the presumed wishes of the demented resident about the end of life, for example, from relatives, during visits or by telephone? yes/no
   2. Do you use any of the following methods to conduct health assessments with dementia residents: admission interview; interview at the end of first month; bi-annual assessment; annual assessment; according to the resident’s state of health
   3. What are the applied methods to conduct the assessments with the relatives: admission interview; interview at the end of first month; bi-annual assessment; annual assessment; according to the resident’s state of health
   4. What procedures do you apply to clarify the presumed wishes of these residents within the health care team: Daily staff meetings within the ward; during medical visit; during interdisciplinary meetings; during supervision meetings
   5. Are there any other procedures applied to clarify the presumed wishes of these residents within the health care team? Please specify: open answer
   6. Among the applied procedures to clarify the presumed wishes of residents within the health care team, which one is the more frequently applied since 2013: daily staff meetings; during the doctor's visit; during interdisciplinary staff meetings; none of these procedures is used more often than the other
   7. Are there any other institutional resources to clarify the presumed wishes of residents in the NH where you are currently that you would like to share? open answer
   8. Are the data on residents' last wishes recorded in the resident's file? yes, always; according to estimated importance; no, never
   9. Are these recorded data reconsidered as the end of life evolves? (only those who have answer “yes, always” or “according to estimated importance” to the previous question): yes/no
   10. Do you have any remark on this topic you would like to communicate? (open answer)
   11. What is the aspect that has most frequently motivated the reconsideration of these instructions since 2013? (persons who have answer “yes, always” to question 9 in this section): new data from the resident; new data from the relatives; new medical data; new data from the spiritual representative; none aspect is mentioned more often than the others
6. **End-of-life decision-making for residents with dementia.** This part concerns decisions made to respect the wishes of demented residents for their end-of-life. Refer in your answers to the end-of-life cases in which you have been involved since 2013. (tick the corresponding box)
   1. What aspects you and your team have made decisions on in end-of-life situations for residents with dementia? Aspects related to religious/spiritual support; aspects related to funeral arrangements; aspects related to healthcare; aspects related to family considerations; aspects related to administrative details (tick all relevant boxes)
   2. Are there any other aspects you have had to make decision on in end-of-life situations for dementia residents since 2013? (open answer)
   3. What aspect has been most frequently submitted to decision making since 2013: Aspects related to religious/spiritual support; aspects related to funeral arrangements; aspects related to healthcare; aspects related to family considerations; aspects related to administrative details; the aspect that I have mentioned in question 2 in this section; none of these aspects was more frequent than other (tick all relevant boxes)
   4. Which of the following issues have you encountered most frequently in relation to the end-of-life situations of residents with dementia since 2013: relatives are reluctant to accept your team's clinical choices; the lack of availability of staff to accompany the dying person as you would like; wills recorded by the dying person that the team considers inadequate to their end-of-life conditions
   5. Among the end-of-life cases of residents with dementia cared for in your NH since 2013, have you used any of the following external resources: mobile psychogeriatric support team; mobile palliative care support team, NH’s medical consultant; ethical advice from an accredited organization, other external resource
   6. If you ticked the "other" box to question 5 in this section, please specify (open answer)
   7. In your opinion, does the terminal care of residents with dementia really take into account their presumed wishes: always; on a regular basis; sometimes; never
   8. How comfortable do you feel about addressing end-of-life issues for dementia residents: comfortable; quite comfortable; not so comfortable; not comfortable at all
   9. Do you find it helpful for end-of-life decisions for dementia residents to have the possibility to refer to a formal advance directive form: very helpful; quite helpful; not so helpful; not helpful at all
   10. Why: (persons who answered “very helpful” to question 9 in this section) (open answer)
   11. Why: (persons who answered “not so helpful” to question 9 in this question) (open answer)
   12. Why: (persons who answered “quite helpful” to question 9 in this question) (open answer)

1. Certificate of Advance Studies [↑](#footnote-ref-1)
2. Diploma of Advance Studies [↑](#footnote-ref-2)
